# Crystallographic orientation-dependent pattern replication in direct imprint of aluminum nanostructures

**DOI:** 10.1186/s11671-015-0788-4

**Published:** 2015-02-28

**Authors:** Ying Yuan, Junjie Zhang, Tao Sun, Cong Liu, Yanquan Geng, Yongda Yan, Peng Jin

**Affiliations:** Center for Precision Engineering, Harbin Institute of Technology, Harbin, 150001 People’s Republic of China; Center of Ultra-precision Optoelectronic Engineering, Harbin Institute of Technology, Harbin, 150001 People’s Republic of China

**Keywords:** Direct imprint, Single-crystalline aluminum, Crystallographic orientation, Nanoindentation, Molecular dynamics

## Abstract

In the present work, we perform molecular dynamics simulations corroborated by experimental validations to elucidate the underlying deformation mechanisms of single-crystalline aluminum under direct imprint using a rigid silicon master. We investigate the influence of crystallographic orientation on the microscopic deformation behavior of the substrate materials and its correlation with the macroscopic pattern replications. Furthermore, the surface mechanical properties of the patterned structures are qualitatively characterized by nanoindentation tests. Our results reveal that dislocation slip and deformation twinning are two primary plastic deformation modes of single-crystalline aluminum under the direct imprint. However, both the competition between the individual deformation mechanisms and the geometry between activated dislocation slip systems and imprinted surface vary with surface orientation, which in turn leads to a strong crystallographic orientation dependence of the pattern replications. It is found that the (010) orientation leads to a better quality of pattern replication of single-crystalline aluminum than the (111) orientation.

## Background

Direct imprint, as one promising variant of nanoimprint lithography technique, is of not only technological significance for the fabrication of functional nanostructures and nanodevices with high resolution and throughput but also fundamental research interests in understanding the properties and deformation behavior of materials at the nanometer scale. By transferring or duplicating patterns on rigid masters into deformable substrates, a variety of structures ranging from a few nanometers to tens of micrometers in dimensional size and with resolution better than 10 nm have been achieved by direct imprint rapidly and inexpensively [1-3]. More importantly, the class of substrate materials that can be dealt by direct imprint is significantly broadened to include semiconductors [1,4] and metals [2,3,5], in addition to polymers [6,7].

The structural patterning in direct imprint is achieved mainly through pressure-induced replication of topographic patterns from rigid masters into substrates by irreversible plastic deformation. Thus, the deformability of substrate materials plays a critical role in determining the quality of patterned structures. It has been demonstrated that although the structures achieved by direct imprint present high global uniformities and fidelities, there are also considerable debris existing in local structures, which may arise from the intrinsic heterogeneous deformation behavior of materials at the nanoscale [8,9]. Since surface integrity greatly affects the performance of functional structures [10], a fundamental understanding of the microscopic deformation mechanisms of materials under direct imprint and their correlation with the macroscopic imprint results is essentially required for the optimization of processing parameters.

Crystallographic orientation is one important parameter that influences deformation behavior of materials because of the anisotropic plasticity, which is governed by activated slip systems. Previous experimental and theoretical work has extensively examined the crystallographic orientation-dependent mechanical response of crystalline materials. For example, it is found that the anisotropic plastic deformation behavior of face-centered-cubic (FCC) metals under tension, compression, bending, indentation, and scratching tests is mainly determined by the activation of {111} < 1-10 > slip systems [11-15]. However, little information about the influence of crystallographic orientation on direct imprint process is known. Moreover, although molecular dynamics (MD) simulation has been widely utilized to explore nanoimprint processes [16-19], there is limited work that evaluates the properties of patterned structures.

Therefore, in the present work, we perform MD simulations to elucidate the underlying deformation mechanisms of single-crystalline aluminum under direct imprint using a rigid silicon master. We further emphasize on the influence of crystallographic orientation on the microscopic deformation behavior of the substrate materials, as well as the macroscopic imprint results in terms of imprint force, topography, and mechanical properties of patterned structures. Furthermore, direct imprint and following nanoindentation experiments are also carried out to validate the theoretical findings by MD simulations.

## Methods

### Simulation method

**Figure 1:**
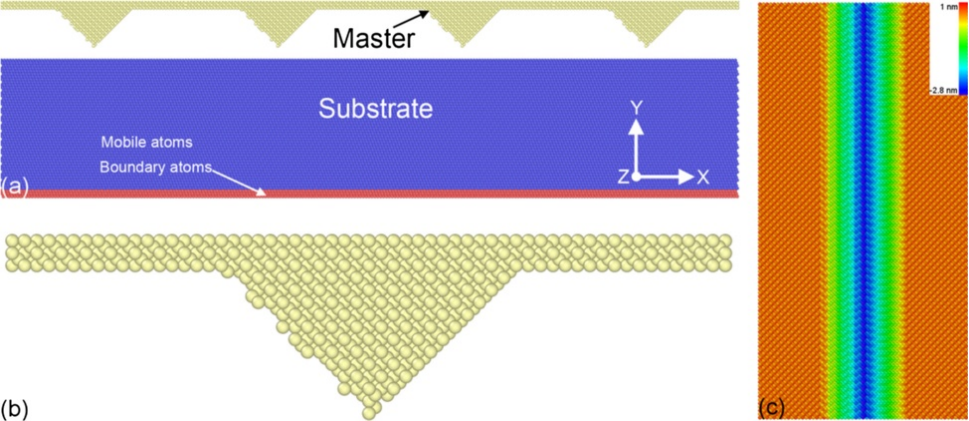
**MD model of direct imprint of Al substrate using Si master. (a)** Atomic configuration of MD model. Atoms are colored according to their virtual types, as red, blue, and yellow colors indicate boundary, mobile, and master atoms, respectively. **(b)** Side view and **(c)** bottom view of single V-shaped tooth in the master. Atoms in (c) are colored according to their atomic heights.

Figure 1a shows that the MD model of direct imprint is composed of a single-crystalline aluminum substrate and a silicon master. The substrate has a dimension of 63, 12, and 32 nm in the *X*, *Y*, and *Z* direction, respectively, and contains approximately 1.5 million atoms. The substrate consists of two kinds of atoms, the boundary atoms that are fixed in space and the mobile atoms which motions follow Newton’s equation of motion, respectively. To examine the influence of crystallographic orientation on the direct imprint process, two single-crystalline aluminum substrates with (010) and (111) free surfaces are considered. The silicon master has four V-shaped teeth on its surface. Figure 1b,c presents the side and bottom views of a single tooth geometry, respectively. The length in *Z* direction and the height in *Y* direction of the tooth are 32 and 3.2 nm, respectively. The angle between the two facets of each tooth is the same as 90°. The silicon master is treated as a rigid body throughout the direct imprint process. The atomic interactions in the Al substrate and that between the Al substrate and the Si master are molded by embedded atom method and Lennard-Jones potential, respectively [19].

The Si master is initially placed above the substrate surface with a distance of 1.2 nm. Prior to the direct imprint, the simulated system is relaxed to its equilibrium configuration at 30 K. The direct imprint process is composed of two sequential stages, the first imprint and following withdrawing, respectively. In the imprint stage, the master moves downwards along the negative *Y* direction to penetrate into the substrate surface with a constant velocity of 20 m/s until a moving distance of 4 nm is reached. After completion of the imprint stage, the master returns to its original position with the same constant velocity of 20 m/s in the following retraction stage. The common neighbor analysis (CNA) is utilized to identify defect types generated in the aluminum substrates [20].

After completion of the direct imprint process, MD simulations of spherical nanoindentation on the patterned surfaces are carried out to characterize the mechanical properties of the patterned structures. The spherical probe with a radius of 3 nm is molded by a strong repulsive potential [21]. The nanoindentation is performed in a displacement-controlled mode by applying a constant velocity of 20 m/s to the probe. We note that a high velocity of 20 m/s is utilized for the MD simulations of both direct imprint and following nanoindentation to make the computational time reasonable for the large-scale simulated systems. All MD simulations of direct imprint and nanoindentation are completed by using the LAMMPS code [22]. The Ovito [23] is utilized to visualize MD data and generate MD snapshots.

### Experimental procedure

**Figure 2:**
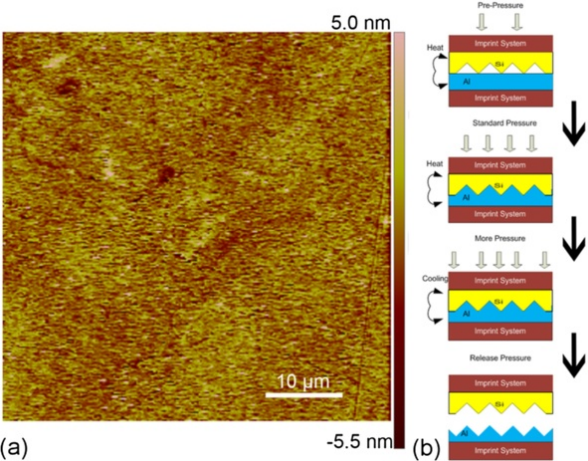
**Configuration of direct imprint experiment. (a)** AFM image of Al(010) surface. **(b)** Schematic illustration of direct imprint procedure: heating at low pressure, increasing pressure at fixed temperature, cooling accompanied with decreasing temperature, and releasing of pressure.

In addition to MD simulations, direct imprint experiments of single-crystalline aluminum substrate using silicon master are also carried out. To be consistent with the MD simulations, single-crystalline Al(010) and (111) surfaces are considered to investigate the effect of crystallographic orientation. The size and the thickness of each substrate are 5 × 5 cm^2^ and 1 cm, respectively. Figure 2a presents the atomic force microscopy (AFM) image of the Al(010) surface, which demonstrates a fine surface roughness of 1 nm.

The Si master has aligned tooth patterns of V-shaped cross section, which is fabricated through anisotropic wet etching technique [24]. The angle between the two facets and the bottom width of each tooth in the Si master is 54.74° and 4 μm, respectively. The height of each tooth is the same as 2.83 μm. The spacing between neighboring teeth is 4 μm.

To achieve the direct imprint of metallic material under accurately controlled pressure and temperature, a home-made direct imprint apparatus is developed. As shown in Figure 2b, direct imprint experiment consists of four sequential stages. In the first stage, the Si master is placed on the Al substrate under a pressure of 1.3 MPa, and the system is heated to 300°C in 20 min. In the second stage, the pressure applied on the master first increases to 4 MPa rapidly and then maintains for 40 min, which enables the pattern duplication from the Si master into the Al substrate. In the third stage, the operating temperature gradually decreases to 130°C in 37 min, accompanied with further increase of the applied pressure to 5.5 MPa. In the final stage, the applied pressure is released thoroughly, and the Si master is removed from the Al substrate.

After completion of the direct imprint, the Al substrates are ultrasonically cleaned in acetone and alcohol solution. To obtain the topographies of the patterned structures, the Al substrates are then scanned by AFM using a silicon tip (RTESP, Bruker Company, Ettlingen, Germany) under the tapping mode. To characterize the mechanical properties of as-imprinted patterned structures, load-controlled nanoindentation tests on the imprinted surfaces are carried out in Agilent Nano Indenter G200 (Agilent Technologies Inc., Santa Clara, CA, USA). A Berkovich diamond tip with a tip radius of 20 nm is utilized for the nanoindentation tests. It should be noted that the MD simulations of either direct imprint or nanoindentation are performed in the displacement-controlled modes, which are different from the load-controlled modes in experiments. Although the discrepancy in methodologies between MD simulations and experiments might cause uncertainties in their quantitative comparison, the MD simulations are powerful to reveal the underlying deformation mechanisms of materials at the nanoscale that cannot be monitored directly by experiments.

## Results and discussion

### On the nature of direct imprint mechanisms

**Figure 3:**
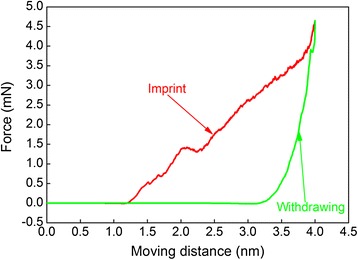
**Force-moving distance curve during the direct imprint simulation of Al(010).**

MD simulations are first conducted to obtain atomistic insights into the direct imprint of single-crystalline aluminum. Figure 3 plots the variation of imprint force with moving distance during the direct imprint of Al(010). In the imprint stage, the force is zero when the master approaches the substrate surface until a moving distance of 1.1 nm is reached, at which the force goes down to a negative value due to the adhesion effect between the master and the substrate. When the master starts to penetrate into the substrate surface, the material first undergoes elastic deformation, accompanied with a rapid increase of the force. However, force-drop phenomenon occurs when the force reaches a local maximum value of 510 nN at a critical moving distance of 1.5 nm, indicating the initiation of plastic deformation in the substrate. Upon further imprint, the force increases continuously with fluctuation phenomena. After completion of the imprint stage, in the following withdrawing stage, the force first decreases precipitously, followed by a minor increase. Then, the force decreases rapidly and becomes zero at a moving distance of 3.2 nm, i.e., the residual imprint depth is 2.0 nm.

**Figure 4:**
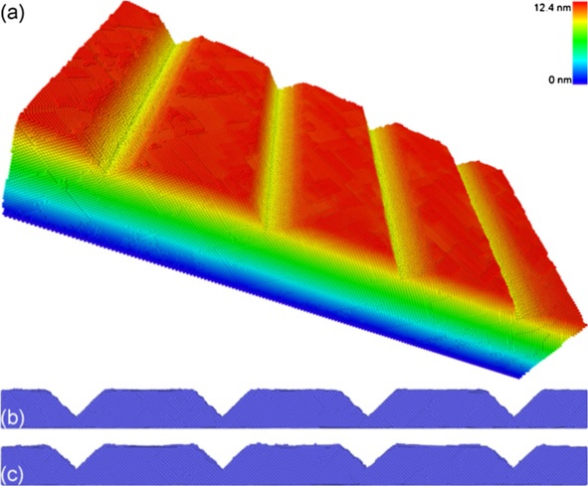
**Patterned structures on Al(010). (a)** Three-dimensional view of patterned structures after the withdrawing stage. Atoms are colored according to their atomic heights. Side view of patterned structure after the **(b)** imprint and **(c)** withdrawing stages.

Figure 4a presents a three-dimensional view of the patterned structures formed on the Al(010) substrate after the direct imprint process. To highlight the patterned structures, atoms are colored according to their atomic heights in the *Y* direction. It is found from Figure 4a that there are four V-shaped concave structures with long-range global uniformity formed on the substrate surface. Figure 4b,c presents the side views of the patterned structures after completion of the imprint and withdrawing stages, respectively. It is found that although there is considerable recovery of the imprinted surface which occurred in the withdrawing stage, the change of cross-sectional profile of the patterned structures is negligible.

**Figure 5:**
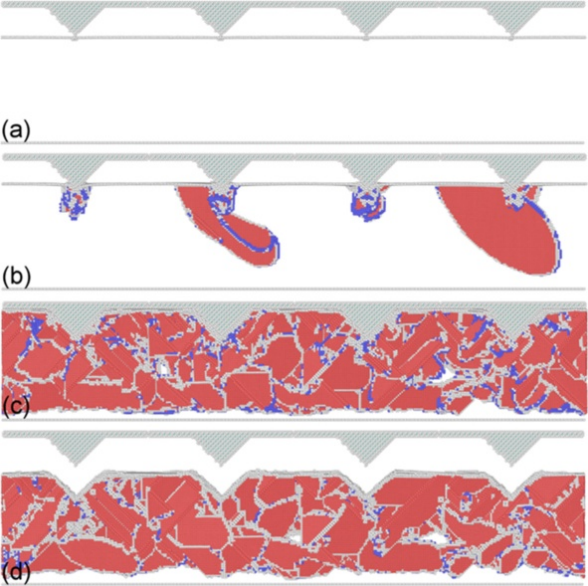
**Defect evolutions during the direct imprint of Al(010).** Instantaneous defect structures at a moving distance of **(a)** 1.32 nm, **(b)** 2.08 nm, and **(c)** 4.0 nm. **(d)** Instantaneous defect structures after completion of the withdrawing stage. Atoms are colored according to their CNA values.

To interpret the characteristics observed in the force-moving distance curves, Figure 5 presents defect evolutions during the direct imprint of the Al(010) substrate. Figure 5a shows that at a moving distance of 1.32 nm, there is no defect generated in the substrate, indicating that the material is undergoing pure elastic deformation. When the applied stress by the master reaches the critical resolve stress of the material, plastic deformation initiates through the nucleation of lattice partial dislocations from the penetrated surface. Dislocation nucleation releases accumulated elastic strain energy, which leads to the decrease of the force shown in Figure 3 [19]. Figure 5b shows that at a moving distance of 2.08 nm, there are defect zones composed of dislocation structures formed beneath each tooth of the master. However, the extent of defect zone beneath each tooth is not uniform with each other. While there are considerable partial dislocations bounced by intrinsic stacking fault formed in the vicinity of the second and fourth teeth, the defect zone is very small for the first and third teeth because of the activation of multiple slip systems. Upon further imprint, nucleated dislocations glide on neighboring {111} slip planes to approach each other, and their multiplication and interaction lead to the formation of sessile and glissile dislocation structures. Furthermore, the average distance between dislocations decreases with increasing dislocation density. Consequently, both dislocations and sessile dislocation structures block the motion of dislocations, which causes significant strain hardening which occurred in the imprinted material [25]. It is known that the deformation ability of ductile metallic materials dominantly depends on the ability of dislocation motion, and the strengthening of the material results in the deterioration of the ductility and the increase of the force, as shown in Figure 3. To accommodate further plastic deformation induced by the movement of the master, successive dislocations emit from the surface and subsequently glide on other two {111} slip planes. And there are mechanical twin boundaries (TBs) formed by the dissociation of partial dislocations, suggesting that deformation twinning is also one important deformation mode of single-crystalline aluminum under the direct imprint [26,27]. Figure 5c shows that the dislocation density within the substrate is high. We note that the fixed bottom may block the propagation of dislocations. Consequently, the force in the imprint stage increases with fluctuations, which are caused by successive nucleation or emission events of dislocations.

In the initial period of the withdrawing stage, the release of applied stress by the master leads to depinning of the pre-existing dislocations generated in the imprint stage, which causes plastic strain recovery accompanied with the decrease of the force. With the withdrawing of the master, part of dislocations move upwards to annihilate at the imprinted surface, which leads to significant surface recovery. The minor increase of the force in the withdrawing stage shown in Figure 3 is caused by the contact of the master with the recovering imprinted surface.

### Influence of crystallographic orientation

**Figure 6:**
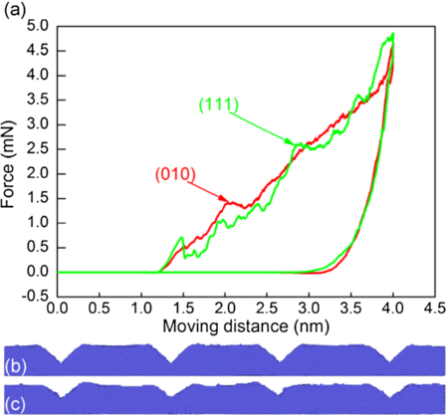
**Direct imprint results of Al(010) and Al(111). (a)** Force-moving distance curves. Side views of patterned structures of **(b)** Al(010) and **(c)** Al(111).

Figure 6a plots variations of the imprint force with moving distance during the direct imprint processes of the two aluminum substrates with different orientations, which demonstrates that the two curves show similar characteristics as described in ‘On the nature of direct imprint mechanisms’ section. However, there are still minor differences existing in the force variations. When the material is undergoing elastic deformation, the (111) orientation shows less compliant response than the (010) orientation because of larger Young’s modulus. The critical force associated with the initiation of plastic deformation is larger for the (111) orientation than that for the (010) orientation, and the corresponding force-drop phenomenon is also more pronounced. It is found from Figure 6 that the (111) orientation has bigger fluctuation of the force than the (010) orientation. Although the (111) orientation has larger maximum force at the largest moving distance, its residual imprint depth is smaller than the (010) orientation.

Figure 6b,c presents the side views of patterned structures after the direct imprint processes of Al(010) and Al(111), respectively. For the (111) orientation, there are also four V-shaped concave structures formed on the substrate surface. However, the deepness of the patterned structures for the (111) orientation is significantly smaller than that for the (010) orientation. More importantly, either the global uniformity or the local surface quality is poorer for the (111) orientation than the (010) orientation. It is found from Figure 6 that while the two sides of the V-shaped concave on the (010) surface show high symmetry, the symmetry is low for the (111) orientation. Furthermore, the machined surface quality of the (010) surface is better than that for the (111) surface.

**Figure 7:**
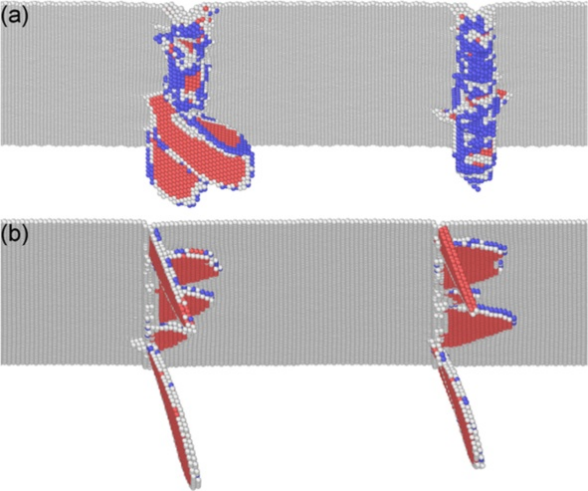
**Incipient plasticity during the direct imprint of Al(010) and Al(111).** Defect structures of **(a)** Al(010) and **(b)** Al(111).

Figure 7a,b presents the instantaneous defect structures at the onset of plasticity of Al(010) and Al(111), respectively. Dynamic inspection of defect evolution shows that the yielding of each substrate is governed by heterogeneous nucleation of dislocations from the imprinted surface, as the {111} slip planes are activated sequentially. It is seen from Figure 7 that there are three and two slip planes activated at the onset of plasticity of the Al(010) and Al(111) orientations, respectively. Moreover, while the activated slip planes have no preferable directions for different teeth, the slip planes for the (111) orientation is the same, as one is 73° inclined and the other is parallel to the (111) free surface. It is well known that there are four {111} slip planes for dislocation motions in FCC crystals. However, the geometry between individual slip planes and imprinted surfaces is different for different orientations, which consequently leads to different material removal behavior.

**Figure 8:**
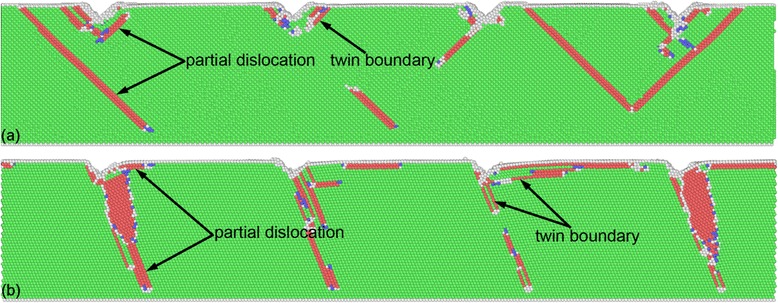
**Cross-sectional views of instantaneous defects in single-crystalline aluminum substrates at a moving distance of 2.5 nm.** Crystallographic orientation: **(a)** (010) and **(b)** (111).

Figure 8a,b presents the cross-sectional views of instantaneous defects in Al(010) and Al (111) at a moving distance of 2.5 nm, respectively. The penetration of the Si master leads to serious plastic deformation occurring in each substrate. Figure 8a shows that for the (010) orientation, dislocation slips dominate the plastic deformation of the material, and deformation twinning is trivial. There are considerable leading partials that are 45° or 135° inclined to the imprinted surface observed in the Al(010) substrate. Moreover, the number of dislocations with different geometries with respect to the (010) imprinted surface is approximately the same with each other, which consequently leads to the symmetrical surface pileup along both sides of the concave grooves. For the (111) orientation, however, Figure 8b shows that lattice partial dislocations are monotonically either inclined or parallel to the imprinted surface, which causes the asymmetrical distribution of the surface pileup. Furthermore, it is seen from Figure 8b that deformation twinning is more pronounced in the Al(111) substrate than that in the Al(010) substrate.

### Mechanical properties of patterned structures

**Figure 9:**
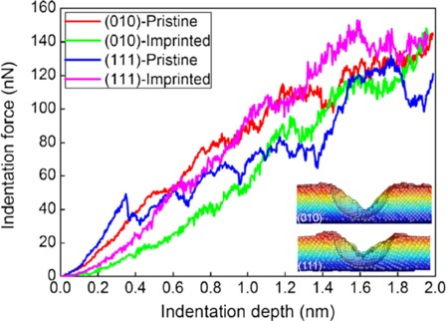
**Indentation force-indentation depth curves during spherical nanoindentation of Al substrates.**

After completion of the direct imprint, MD simulations of spherical nanoindentation are performed to characterize the mechanical properties of the patterned structures. In addition to the imprinted surfaces, pristine surfaces with different orientations are also considered. Figure 9 plots the indentation force-indentation depth curves for the imprinted and pristine surfaces. The inset in Figure 9 shows the cross-sectional profile of the indented patterns for the imprinted (010) and (111) surfaces, which indicates that nanoindentation is performed on the center of the V-shaped concave for each substrate. For the nanoindentation of pristine surface, the material first undergoes elastic deformation accompanied with rapid increase of the indentation force. Young’s modulus derived from the elastic response according to Hertzian contact theory is 76 and 98 GPa for (010) and (111) GPa, respectively. The Poission ratios for the aluminum substrate and diamond probe are 0.35 and 0.07, and Young’s modulus for the rigid diamond probe is 1140 GPa [25]. And the critical force at the yield point is 20 and 50 nN for (010) and (111) GPa, respectively.

Figure 9 shows that both Young’s modulus and critical force is lower for the patterned structures than that for the pristine surface. The derived Young’s modulus for the patterned Al(010) and Al(111) is 40 and 56 GPa, respectively. While the yielding of pristine surface is achieved by the nucleation of lattice dislocations from indented surface, the plastic deformation of patterned surface is initiated by the motion of the pre-existing dislocations [25,28]. For the (010) orientation, the indentation force for the patterned structure is smaller than that for the pristine surface through the indentation test. The indentation force in the elastic deformation of the patterned Al(111) surface is lower than the pristine surface. However, the indentation force of the patterned Al(111) surface increases to higher than the pristine surface in the following plastic deformation.

### Direct imprint experiment

**Figure 10:**
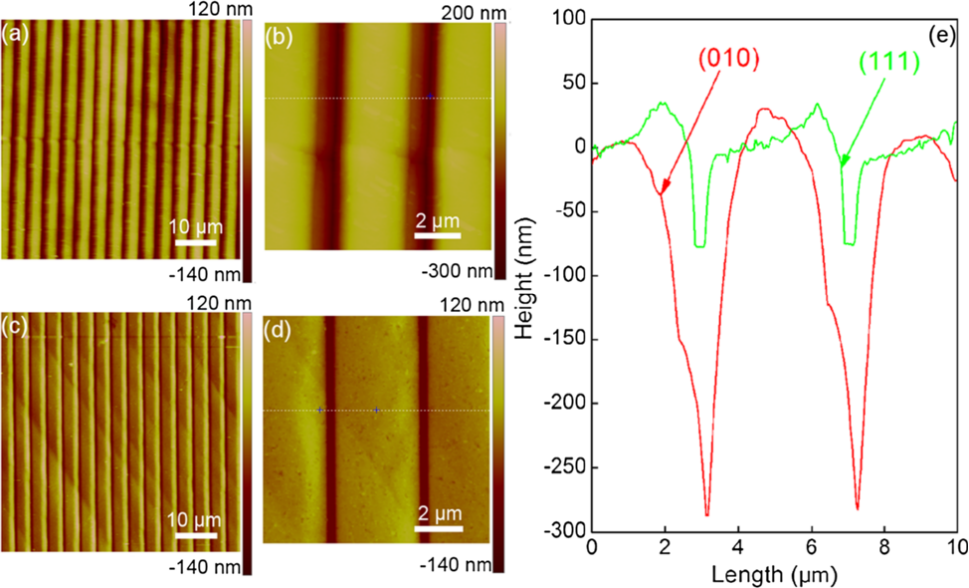
**Direct imprint results.** AFM images of patterned structures for **(a)** (010) orientation and **(c)** (111) orientation. Enlarged view for **(b)** (010) orientation and **(d)** (111) orientation. **(e)** The cross-sectional profiles in **(b)** and **(d)**.

In addition to MD simulations, direct imprint experiments are also carried out on single-crystalline Al substrates with different surface orientations. Figure 10a,c presents AFM images of the patterned structures on the Al(010) and Al(111) surfaces, respectively. It is seen from Figure 10 that for each surface orientation, there are aligned concave groove patterns formed on the sample surface, accompanied with surface pileup residing on both sides of the groove. However, both the groove width and the volume of surface pileup are larger for the (010) orientation than that for the (111) orientation. Figure 10b,d respectively shows the enlarged views of representative zones in Figure 10a,c, and Figure 10e further plots their cross-sectional profiles. It is found from Figure 10e that for each surface orientation, the spacing between each concave groove is the same as 4 μm, which is consistent with the geometry of the Si master. However, the geometry of individual grooves is highly dependent on the surface orientation. For the (010) orientation, Figure 10e shows that the groove depth is close to 300 nm of the tooth height of the Si master, indicating that the plastic recovery during the withdrawing stage is negligible. Furthermore, the surface pileup shows high symmetry with respect to the groove. However, the groove depth for the (111) orientation is 88 nm, and the symmetry of the surface pileup is deteriorated.

**Figure 11:**
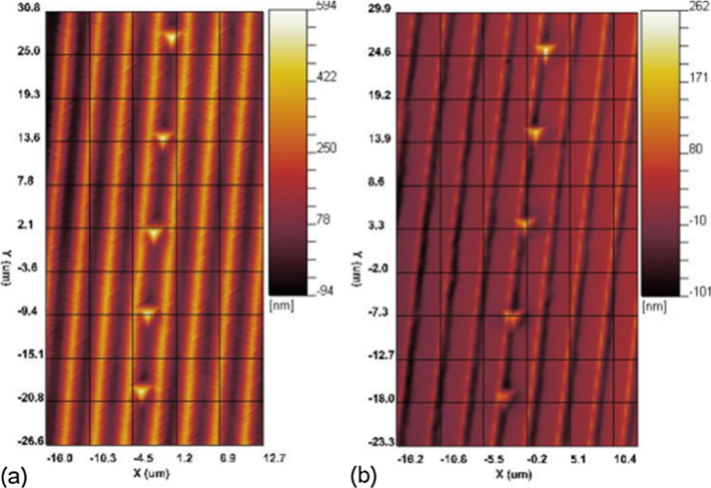
**Nanoindentation tests on the patterned structures.** Crystallographic orientation: **(a)** (010) and **(b)** (111).

After completion of the direct imprint, the patterned structures are subjected to load-controlled nanoindentation tests. To characterize the mechanical properties of the imprinted surface, nanoindentation tests are performed on the concave surface of the patterned structures. For individual concave grooves, five nanoindentation tests are carried out. For comparison purpose, nanoindentation tests on the pristine surfaces are also considered. Figure 11a,b presents the nanoindentation results on the patterned structures for the (010) and (111) orientations, respectively. And Table 1 summarizes the mechanical properties, in terms of Young’s modulus and hardness, of the Al substrates. It is found that for each surface orientation, the mechanical properties of the patterned surface are deteriorated, due to the existence of defects generated during the direct imprint process. And both the Young’s modulus and the hardness for the (111) orientation are larger than that for the (010) orientation. It indicates that the experimental results of direct imprint and following nanoindentation qualitatively agree well with the observations from MD simulations.

**Table 1:** **Mechanical properties of Al substrates with different surface orientations**

	**(010)-pristine**	**(010)-imprinted**	**(111)-pristine**	**(111)-imprinted**
Young’s modulus (GPa)	65.3	59.0	76.3	64.0
Hardness (MPa)	221	207	331	308

## Conclusions

In summary, we perform MD simulations and experiments to elucidate the underlying deformation mechanisms of single-crystalline aluminum under the direct imprint using a silicon master. The influence of crystallographic orientation on the direct imprint is further examined. It is found that the plastic deformation of single-crystalline aluminum is governed by dislocation activities and deformation twinning in parallel. And the existence of lattice defects leads to the deterioration of mechanical properties of the patterned structures, as compared to the pristine surface. It is found that both the microscopic deformation behavior and the macroscopic pattern replication have strong dependence on the crystallographic orientation. Either the depth or the width of the concave grooves is larger for the (010) orientation than that for the (111) orientation. Furthermore, the (010) orientation leads to a better surface quality of the patterned structures than that for the (111) orientation.
